# Provider beliefs in effectiveness and recommendations for primary HPV testing in 3 health-care systems

**DOI:** 10.1093/jncics/pkac086

**Published:** 2022-12-05

**Authors:** Gina R Kruse, Jacquelyn M Lykken, Eric J Kim, Jennifer S Haas, Robin T Higashi, Steven J Atlas, Anne Marie McCarthy, Jasmin A Tiro, Michelle I Silver, Celette S Skinner, Aruna Kamineni

**Affiliations:** Division of General Internal Medicine, Massachusetts General Hospital, Harvard Medical School, Boston, MA, USA; Department of Population and Data Sciences, University of Texas Southwestern Medical Center, Dallas, TX, USA; Department of Population and Data Sciences, University of Texas Southwestern Medical Center, Dallas, TX, USA; Division of General Internal Medicine, Massachusetts General Hospital, Harvard Medical School, Boston, MA, USA; Department of Population and Data Sciences, University of Texas Southwestern Medical Center, Dallas, TX, USA; Harold C. Simmons Comprehensive Cancer Center, University of Texas Southwestern Medical Center, Dallas, TX, USA; Division of General Internal Medicine, Massachusetts General Hospital, Harvard Medical School, Boston, MA, USA; Department of Biostatistics, Epidemiology and Biostatistics, Perelman School of Medicine, University of Pennsylvania, Philadelphia, PA, USA; Department of Public Health Sciences, University of Chicago—Biological Sciences Division, Chicago, IL, USA; Division of Public Health Sciences, Department of Surgery, Washington University School of Medicine, St Louis, MO, USA; Department of Population and Data Sciences, University of Texas Southwestern Medical Center, Dallas, TX, USA; Harold C. Simmons Comprehensive Cancer Center, University of Texas Southwestern Medical Center, Dallas, TX, USA; Kaiser Permanente Washington Health Research Institute, Seattle, WA, USA

## Abstract

In 2018, the US Preventive Services Task Force endorsed primary human papillomavirus testing (pHPV) for cervical cancer screening. We aimed to describe providers’ beliefs about pHPV testing effectiveness and which screening approach they regularly recommend. We invited providers who performed 10 or more cervical cancer screens in 2019 in 3 healthcare systems that had not adopted pHPV testing: Kaiser Permanente Washington, Mass General Brigham, and Parkland Health; 53.7% (501/933) completed the survey between October and December 2020. Response distributions varied across modalities (*P* < .001), with cytology alone or cotesting being more often viewed as somewhat or very effective for 30- to 65-year-olds compared with pHPV (cytology alone 94.1%, cotesting 96.1%, pHPV 66.0%). In 21- to 29-year-olds, the pattern was similar (cytology alone 92.2%, 64.7% cotesting, 50.8% pHPV). Most providers were either incorrect or unsure of the guideline-recommended screening interval for pHPV. Educational efforts are needed about the relative effectiveness and recommended use of pHPV to promote guideline-concordant care.

In 2018, the United States Preventive Services Task Force (USPSTF) updated their cervical cancer screening guidelines by adding 5-yearly primary HPV testing (pHPV) for women aged 30-65 years as an alternative to 3-yearly cytology or 5-yearly cotesting (cytology coupled with HPV testing).

Alignment between provider beliefs and recommendations and guideline-concordant care may improve decision quality and avoid misuse and overuse of screening tests (1,2). Our objectives were to describe provider beliefs in the effectiveness of USPSTF guideline-based screening strategies on reducing cervical cancer mortality and which approach (modality and interval) they regularly recommend for women in different age groups.

We conducted this study within 3 health-care systems that had not implemented pHPV testing at the time of the survey: Kaiser Permanente Washington; Mass General Brigham; and Parkland Health, with academic oversight from University of Texas Southwestern Medical Center. Kaiser Permanente Washington is a mixed-model health-care system providing care and coverage in Washington State (3). Mass General Brigham is a Boston area health-care system. Parkland Health, with academic oversight from University of Texas Southwestern Medical Center is a safety-net health-care system for Dallas County residents (3). Institutional review boards at each site approved study activities.

We identified providers who performed at least 10 cervical cancer screening tests during 2019 with designated specialties of internal medicine, family medicine, or obstetrics/gynecology (ob/gyn). Eligible providers (n = 933) were asked to complete a confidential, web-based survey from October 2020 to December 2020.

Survey content was adapted from earlier provider surveys of cancer screening beliefs and recommendations (Supplementary Methods, available online) (4,5). Provider sociodemographic measures included age, gender identity, provider type, specialty, full-time or part-time employment status, years in current practice, weekly patient volume, and practice size. Providers were asked to rate their beliefs in the effectiveness of USPSTF-recommended screening tests at reducing lifetime risk of cervical cancer mortality separately for average-risk women aged 21-29 years and 30-65 years. Five response options were offered: not very effective (<20% reduction in cervical cancer deaths), somewhat (20-50%), very (>50%), effectiveness not known, and I am not sure. They were further asked to indicate which screening approach (modality and interval) they regularly recommend for women in different age groups; for providers who indicated that pHPV was currently unavailable in their practice, they were asked whether and how they would recommend pHPV if it became available.

We compared distributions of provider responses regarding beliefs about the effectiveness of screening by age group across pHPV, cytology only, and cotesting modalities, and their recommended screening approach by age group across health-care systems using Pearson’s χ^2^ tests (alpha = .05). Analyses were conducted using SAS version 9.4.

Of 933 eligible providers, 501 (53.7%) completed the survey, and 9 respondents were excluded because they had not performed a cervical cancer screen within 12 months of survey administration, leaving 492 available for analysis. Characteristics of eligible respondents and those who did not respond are shown in Supplementary Table 1 (available online).

Overall, 70.5% of respondents were physicians (MD or DO), 20.3% were advanced practice nurses (NP or CNM), and 9.1% were physician assistants (Supplementary Table 1, available online). Specialty distribution was 40.0% family medicine, 34.4% internal medicine, and 25.6% ob/gyn.

Belief in effectiveness varied across screening modalities (Figure 1). Across sites, pHPV was viewed as very effective for 21- to 29-year-olds among one-third of providers (31.1%); in contrast, 56.6% of providers viewed it as very effective for 30- to 65-year-old women. For 21- to 29-year-old women, cotesting was viewed as very effective by 33.8% and cytology alone (with reflex HPV testing for Atypical squamous cells of undetermined signficance [ASC-US] results) was viewed as very effective by 61.9% of providers. For 30- to 65-year-old women, cytology alone was believed to be very effective by a similar proportion of providers (63.5%), and cotesting in this age group was viewed as very effective by most (90.2%).

**Figure 1.**
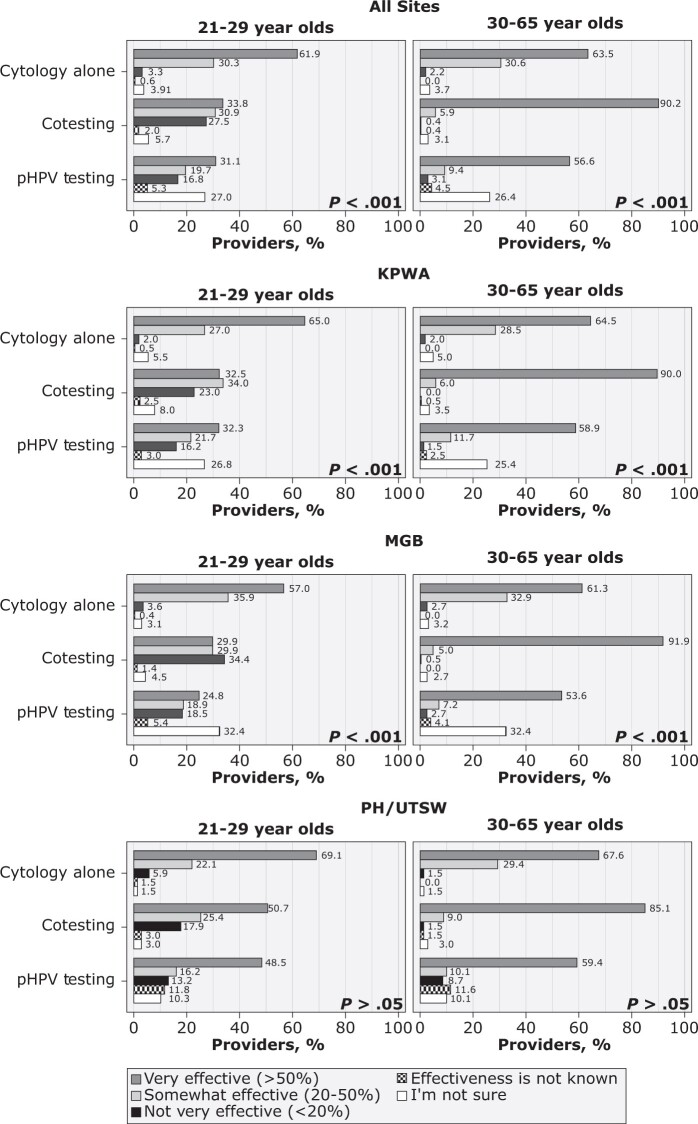
Distribution of provider beliefs on the effectiveness of the use of primary human papillomavirus (pHPV) testing, cytology alone, and cotesting on reducing lifetime risk of cervical cancer mortality by age and site. Providers who did not respond to this survey item for each age category were excluded (cytology alone, for patients 21-29 years old, n = 1 and for patients 30-65 years old, n = 0; cotesting, for patients 21-29 years old, n = 4 and for patients 30-65 years old, n = 4; and primary HPV testing, for patients 21-29 years old, n = 4 and for patients 30-65 years old, n = 4). The χ^2^*P* values at the **lower right corner** of each section compare provider responses across screening modalities (α = .05). KPWA = Kaiser Permanente Washington; MGB = Mass General Brigham; PH/UTSW = Parkland Health/University of Texas Southwestern Medical Center.

Across sites, most providers were incorrect or unsure of the guideline-recommended interval for pHPV. The most common selection for pHPV screening interval was “I am not sure” (39.7% for 21- to 29-year-olds; 37.8% for 30- to 65-year-olds). Only 31.9% selected the guideline-concordant 5-year interval for 30- to 65-year-olds. In contrast, 75.8% recommended cytology-based tests at guideline-concordant intervals in 30- to 65-year-olds, including 5-year interval for cotesting (72.9%) or 3-year interval for cytology alone (2.9%). Regular screening recommendations for 21- to 29-year-olds followed a similar pattern: 15.2% selected that primary pHPV is *not* recommended, whereas 84.4% selected the guideline-concordant 3-year interval for cytology alone. The distribution of screening recommendations varied by site (Figure 2).

**Figure 2.**
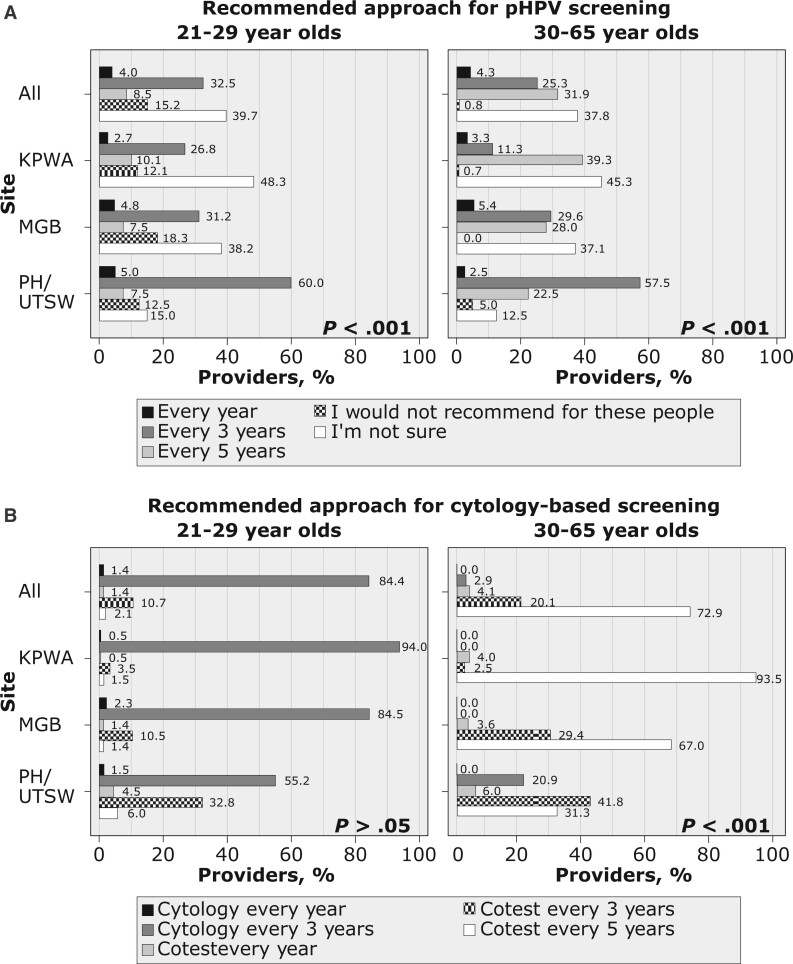
Distribution of providers’ regular recommendation for cervical cancer screening by modality (primary human papillomavirus [pHPV] screening vs cytology-based screening), age, and site. (**A**) Approach regularly recommended by provider for cervical cancer screening using pHPV testing if it were to become available at their practice among those who indicated that this modality was not currently available (n = 375), with providers who did not respond to this survey item for each age category excluded (patients 21-29 years old, n = 4; 30-65 years old, n = 3). (**B**) Approach regularly recommended by provider for cervical cancer screening using cytology alone or cotesting, with providers who did not respond to this survey item for each age category excluded (patients 21-29 years old, n = 6; 30-65 years old, n = 6). The χ^2^*P* values at the **lower right corner** of each section compare provider responses across sites (α = .05). KPWA = Kaiser Permanente Washington; MGB = Mass General Brigham; PH/UTSW = Parkland Health/University of Texas Southwestern Medical Center.

Our study of providers, who deliver cervical cancer screening in 3 diverse US health-care systems, found large gaps in providers’ knowledge of the effectiveness of pHPV and the guideline-recommended screening interval for this modality. Fewer providers believed in the effectiveness of pHPV to reduce cervical cancer mortality and were less likely to recommend pHPV in the correct age group and screening interval compared with cytology-based screening modalities.

The health-care systems in this study, like most US health-care systems, had not implemented pHPV testing at the time of survey administration in 2020. Although the updated USPSTF guidelines were published in 2018, pHPV remains uncommon (6). Lack of experience with pHPV may result in lower awareness of its effectiveness. However, provider knowledge of screening test effectiveness ideally should be based on evidence rather than individual practice experience, and providers of cervical cancer screening should be knowledgeable about all guideline-recommended options for their patients.

The limitations we observed in provider knowledge of pHPV for 21- to 29-year-olds may be due, in part, to differences in guidelines. For example, the American Cancer Society recommends pHPV in 25- to 29 year-olds, but the USPSTF does not (7). We also did not examine surveillance testing beliefs and recommendations from the American Society for Colposcopy and Cervical Pathology (8). Another limitation of our study is that although the identical survey was implemented, respondents at different sites may have interpreted questions differently due to local protocols and context.

In the United States, pHPV will likely become the preferred cervical cancer screening strategy due to its greater sensitivity compared with cytology alone and lower cost than cotesting (9). Ensuring providers are knowledgeable about screening test effectiveness is necessary for them to successfully partner with patients to make informed screening decisions. Our study suggests that efforts are needed to increase provider knowledge of pHPV effectiveness and guideline-recommended cervical cancer screening care.

## Supplementary Material

pkac086_Supplementary_Data

## Data Availability

The data underlying this article are available in the article and in its online supplementary material. Information about data sharing in the METRICS project are provided at: https://healthcaredelivery.cancer.gov/prospr/datashare.html.

## Notes

**Role of the funder:** The funder had no role in the design of the study; the collection, analysis, and interpretation of the data; the writing of the manuscript; or the decision to submit the manuscript for publication.

**Author disclosures:** GRK has a family financial interest in a health IT company, Dimagi, Inc. All other authors report no disclosures.

**Author contributions:** GRK-conceptualization, methodology, writing original draft; JML-methodology, formal analysis, data curation, writing review and editing; EJK-methodology, data curation, writing review and editing, JSH-conceptualization, funding acquisition, supervision, writing review and editing; RTH-conceptualization, writing review and editing, SJA-conceptualization, writing review and editing; AMM-conceptualization, writing review and editing; JAT-conceptualization, supervision, funding acquisition, writing review and editing; MIS-conceptualization, writing review and editing; CSS-conceptualization, writing review and editing, supervision, AK-conceptualization, writing original draft, methodology, supervision, funding acquisition.

**Prior presentations:** The main results in this manuscript were presented as a poster at the Society for General Internal Medicine Annual Meeting on April 7, 2022 in Orlando, FL.

**Acknowledgements:** The authors wish to thank the participating MultilEvel OpTimization of the CeRvIcal Cancer Screening Process in Diverse Settings and Populations (METRICS) sites for the data provided for this study. A description of the METRICS study, investigators, and staff are available at https://utsouthwestern.edu/labs/prospr-metrics/about/team.html.
