# Supplementary material for: Provider beliefs in effectiveness and recommendations for primary HPV testing in 3 health-care systems
Source: JNCI Cancer Spectr. 2022 Dec 5;7(1):pkac086. doi: 10.1093/jncics/pkac086 (PMC9825247; doi:10.1093/jncics/pkac086)
Supplement: pkac086_Supplementary_Data [file pkac086_supplementary_data.pdf]

Supplementary Methods

A1. Screening tests are those done in the absence of any symptoms or signs of disease. How ***effective*** do you believe the following screening tests are in reducing **deaths from cervical cancer**?

	Not Very Effective(<20% reduction)	Somewhat effective (20 to 50% reduction)	Very Effective (>50% reduction)	Effectiveness not known	I am not sure
Pap test alone (with reflex HPV test for ASC-US results) for age:					
<21 years	□	□	□	□	□
21-29 years	□	□	□	□	□
30-64 years	□	□	□	□	□
65+ years	□	□	□	□	□
Pap and HPV co-testing for age:					
<21 years	□	□	□	□	□
21-29 years	□	□	□	□	□
30-64 years	□	□	□	□	□
65+ years	□	□	□	□	□
Primary HPV testing *(i.e., clinician-performed HPV testing with a reflex Pap if HPV+)* for age:					
<21 years	□	□	□	□	□
21-29 years	□	□	□	□	□
30-64 years	□	□	□	□	□
65+ years	□	□	□	□	□

A2. A unique aspect of cervical cancer prevention is that we have a vaccine to prevent infection with high-risk HPV types. How effective do you believe **HPV vaccination** is in reducing **new cases** of cervical cancer? *(Select one answer for each age group.)*

	Not Very Effective (<20% reduction)	Somewhat Effective (20-50% reduction)	Very Effective (>50% reduction)	Effectiveness not known	I am not sure
If vaccine is administered to the following age groups:					
≤ 14 years	☐	☐	☐	☐	☐
15-26 years	☐	☐	☐	☐	☐
27-45 years	☐	☐	☐	☐	☐
46-64 years	☐	☐	☐	☐	☐
65+ years	☐	☐	☐	☐	☐

B1. During a typical week, approximately how many female patients do you see?

☐ 25 or fewer

☐ 26-50

☐ 51-75

☐ 76-100

☐ 101-125

☐ More than 125

B2. In the past month, have you personally performed a Pap test, Pap and HPV co-test, or primary HPV test?

☐ Yes

☐ No

B3. **In the past month**, have you been responsible for following-up your female patients' Pap and/or HPV test results even if you didn't perform the test yourself?

- ☐ Yes
- ☐ No

B4. Is primary HPV testing (i.e., clinician-performed HPV testing with a reflex Pap if HPV+), available as an option in your practice?

- ☐ Yes -> Program survey to ask Questions B5 – B6, then skip to B9
- ☐ No -> Program survey to ask Questions B7 – B8, then proceed to B9

B5. For **routine cervical cancer screening** in each age group, which of the following approaches do you **regularly recommend** in your practice? *(Select one answer for each age group.)*

	Pap alone every year	Pap alone every 3 years	Pap & HPV co-testing every year	Pap & HPV co-testing every 3 years	Pap & HPV co-testing every 5 years	Primary HPV testing every year	Primary HPV testing every 3 years	Primary HPV testing every 5 years	I do not recommend screening for these women
For women ages: <21 years	☐	☐	☐	☐	☐	☐	☐	☐	☐
21-29 years	☐	☐	☐	☐	☐	☐	☐	☐	☐
30-65 years	☐	☐	☐	☐	☐	☐	☐	☐	☐
>65 years	☐	☐	☐	☐	☐	☐	☐	☐	☐

B6. ***Among women in each age group,*** for what percentage do you **perform** primary HPV testing? *(Select one answer for each age group.)*

	0%	1 – 25%	26 – 50%	51 – 75%	76 – 100%
For women ages: <21 years	□	□	□	□	□
21-29 years	□	□	□	□	□
30-65 years	□	□	□	□	□
>65 years	□	□	□	□	□

B7. For **routine cervical cancer screening** in each age group, which of the following approaches do you **regularly recommend** in your practice?

(Select one answer for each age group.)

	Pap alone every year	Pap alone every 3 years	Pap & HPV co-testing every year	Pap & HPV co-testing every 3 years	Pap & HPV co-testing every 5 years	I do not perform cervical cancer screening for these women
For women ages: <21 years	□	□	□	□	□	□
21-29 years	□	□	□	□	□	□
30-65 years	□	□	□	□	□	□
>65 years	□	□	□	□	□	□

B8. ***If primary HPV testing becomes available in your practice***, how often would you recommend it as a screening option for women in each of these age groups? *(Select one answer for each age group.)*

	Every year	Every 3 years	Every 5 years	I would not recommend for these women	I am not sure
For women ages: <21 years	□	□	□	□	□
21-29 years	□	□	□	□	□
30-65 years	□	□	□	□	□
>65 years	□	□	□	□	□

H1. Do you work full time or part time? Please include all of your work activities (e.g., research, administration), not just clinical activities.

- ☐ Full time
- ☐ Part time

H2. What is your degree or certification?

- ☐ Physician (MD or DO)
- ☐ Nurse Practitioner or Nurse Midwife
- ☐ Physician Assistant
- ☐ Other If other, please specify: ______________________

H3. What is your specialty?

- ☐ Family Medicine
- ☐ General Internal Medicine
- ☐ Obstetrics and Gynecology
- ☐ Gynecology Oncology
- ☐ Other If other, please specify: ______________________

H4. What is your current employment arrangement?

- ☐ Employed by a hospital
- ☐ Employed by a health plan
- ☐ Employed by a medical group
- ☐ Practice owner/partner/associate
- ☐ Other If other, please specify: ______________________

H5. What is your gender identity?

☐ Woman

☐ Man

☐ Non-binary

☐ Prefer to self-describe as: ________________________________-

☐ Prefer not to answer

H6. What is your age?

☐ Less than 30 years old

☐ 30-39 years old

☐ 40-49 years old

☐ 50-59 years old

☐ 60-69 years old

☐ 70 years old or older

☐ Prefer not to state

H7. Do you practice in or have an affiliation with an academic medical center?

☐ Yes

☐ No

H8. How long have you worked in your current practice?

- ☐ Less than a year
- ☐ 1 year to less than 3 years
- ☐ 3 years to less than 5 years
- ☐ 5 years to less than 10 years
- ☐ 10 years or more

H9. How many physicians are at the main clinic where you practice?

- ☐ 1-10
- ☐ 11-30
- ☐ 31-50
- ☐ 51 or more

Supplementary Table 1. Characteristics of providers who responded and were eligible for the survey and those who were either ineligible or did not complete the survey, overall and by healthcare system

Provider Characteristics[1,2]	KPWA n=414		MGB n=503		PH/UTSW n=186		Total n=1,103	
Eligible and responded to survey?	Yes	No	Yes	No	Yes	No	Yes	No
	n=200 (48.3%)	n=214 (51.7%)	n=223 (44.3%)	n=280 (55.7%)	n=69 (37.1%)	n=117 (62.9%)	N=492 (44.6%)	N=611 (55.4%)
	n (%)	n (%)	n (%)	n (%)	n (%)	n (%)	n (%)	n (%)
Age (years)								
< 40	89 (45.6)	101 (47.2)	51 (23.2)	57 (21.8)	21 (31.3)	33 (28.2)	161 (33.4)	191 (32.3)
41-49	59 (30.3)	43 (20.1)	54 (24.6)	70 (26.8)	18 (26.9)	33 (28.2)	131 (27.2)	146 (24.7)
50-59	26 (13.3)	39 (18.2)	71 (32.3)	75 (28.7)	18 (26.9)	24 (20.5)	115 (23.9)	138 (23.3)
60+	21 (10.8)	31 (14.5)	44 (20.0)	59 (22.6)	10 (14.9)	27 (23.1)	75 (15.6)	117 (19.8)
Prefer Not to State / Unknown	5	0	3	19	2	0	10	19
Gender Identity								
Women	154 (79.4)	164 (76.6)	193 (88.5)	219 (78.2)	61 (89.7)	97 (82.9)	408 (89.7)	480 (78.6)
Men	40 (20.6)	50 (23.4)	25 (11.5)	61 (21.8)	7 (10.3)	20 (17.1)	72 (10.3)	131 (21.4)
Other / Unknown	6	0	5	0	1	0	12	0
Provider Type								
Physician	143 (71.5)	163 (76.2)	184 (82.5)	252 (90.0)	20 (29.0)	68 (58.1)	347 (70.5)	483 (79.1)
Nurse Practitioner / Nurse Midwife	27 (13.5)	9 (4.2)	24 (10.8)	9 (3.2)	49 (71.0)	49 (41.9)	100 (20.3)	67 (11.0)
Physician's Assistant	30 (15.0)	42 (19.6)	15 (6.7)	19 (6.8)	0 (0.0)	0 (0.0)	45 (9.1)	61 (10.0)
Provider Specialty								
Family Medicine	147 (73.5)	167 (78.0)	21 (9.9)	14 (5.0)	28 (40.6)	35 (29.9)	197 (40.0)	216 (35.4)
General Internal Medicine	12 (6.0)	22 (10.3)	156 (69.5)	147 (52.5)	2 (2.9)	19 (16.2)	169 (34.4)	188 (30.8)
Ob/gyn / Gyn onc	41 (20.5)	25 (11.7)	46 (20.6)	119 (42.5)	39 (56.5)	63 (53.9)	126 (25.6)	207 (33.9)
Years in Current Practice								
< 3	62 (31.0)	74 (34.6)	27 (12.3)	49 (19.9)	19 (27.9)	105 (90.5)	108 (22.1)	228 (39.6)
3- < 5	34 (17.0)	18 (8.4)	28 (12.7)	33 (13.4)	10 (14.7)	3 (2.6)	72 (14.8)	54 (9.4)
5- < 10	43 (21.5)	38 (17.8)	45 (20.5)	39 (15.9)	17 (25.0)	2 (1.7)	105 (21.5)	79 (13.7)
≥ 10	61 (30.5)	84 (39.3)	120 (54.6)	125 (50.8)	22 (32.4)	6 (5.2)	203 (41.6)	215 (37.3)
Unknown	0	0	3	34	1	1	4	35

Abbreviations: KPWA, Kaiser Permanente Washington; MGB, Mass General Brigham; PH/UTSW, Parkland Health – University of Texas Southwestern Medical Center; Ob/gyn, obstetrics and gynecology; and Gyn onc, gynecology oncology.

[1]Providers eligible for the survey at each healthcare system included those who performed a minimum of 10 cervical cancer screening tests during 2019, had a designated specialty of general internal medicine, family medicine, or obstetrics/gynecology, and were physicians, physician assistants, certified nurse midwives, or nurse practitioners. Note that more providers were eligible to receive the survey than the survey was ultimately sent to due to contact restrictions at sites, which led to the higher response rate noted in the Methods.

[2] Employment status, number of physicians in practice, and the typical number of female patient office visits per week were not available for these providers. Providers that completed the survey performed more Pap tests in 2019 than providers that did not complete the survey (median [IQR] patients tested in 2019 for KPWA, 57 [33,89] vs. 41.5 [22, 88]; MGB, 53 [29, 109] vs. 40.5 [19, 82.5]; PH/UTSW 131 [25, 429] vs. 67 [24, 182]; and overall, 57 [30, 105] vs. 44 [21, 95]).
